# Clinical efficacy of probiotics in improving intestinal mucosal permeability for functional dyspepsia patients with anxiety

**DOI:** 10.3389/fmicb.2025.1614705

**Published:** 2025-06-18

**Authors:** Kang-ming Huang, Hua-bin Qiu, Dong-ping Liao, Ya-yue Jiang, Ying-Han Deng, Hong-bin Chen

**Affiliations:** ^1^Department of Gastroenterology, Sanming First Hospital Affiliated to Fujian Medical University, Sanming, China; ^2^Department of Endoscopy Room, Sanming First Hospital Affiliated to Fujian Medical University, Sanming, China; ^3^Department of Nephrology, Sanming First Hospital Affiliated to Fujian Medical University, Sanming, China

**Keywords:** probiotics, anxiety, functional dyspepsia, brain-gut axis, intestinal mucosal permeability

## Abstract

**Background:**

The aim of this study was to investigate whether probiotics are effective in improving symptoms of functional dyspepsia (FD) accompanied by anxiety.

**Methods:**

There were 116 patients with FD accompanied by anxiety and 114 patients without anxiety. Each group was randomly divided into an intervention group and a control group. The intervention group received probiotics in addition to conventional acid suppression and gastric protection, as well as prokinetic treatments. The control group received conventional treatment plus a placebo.

**Results:**

Before treatment, significant differences were observed in IL-1, IL-6, TNF-*α*, LPS, Zonulin, DAO, and I-FABP between patients with FD accompanied by anxiety and those without anxiety (*p* < 0.05). Following the intervention with probiotics, the group with FD and anxiety experienced significant decreases in IL-1, IL-6, TNF-*α*, LPS, Zonulin, and DAO (*p* < 0.001), as well as significant reductions in the Patient Assessment of Gastrointestinal Disorders-Symptom Severity Index (PAGI-SYM) and the Hamilton Anxiety Scale (HAMA) scores (*p* < 0.001). In contrast, the intervention group with FD but without anxiety had significant reductions in IL-6 and TNF-*α* (*p* < 0.05), along with a significant decrease in the PAGI-SYM score (*p* < 0.001). Binary logistic regression analysis further revealed that lower I-FABP values (OR = 0.999, *p* = 0.036), lower LPS values (OR = 0.998, *p* = 0.013), and probiotic intervention (OR = 5.138, *p* = 0.000) were significantly associated with symptom relief.

**Conclusion:**

The intervention significantly improved the symptoms and anxiety scores of patients with FD and anxiety, and the changes in intestinal mucosal permeability indexes were closely related to symptom relief and anxiety improvement, Probiotic interventions may be an effective means of improving symptoms in patients with FD.

**Clinical trial registration:**

identifier ChiCTR2300077847 https://www.chictr.org.cn/.

## Introduction

Functional Dyspepsia (FD) is a common functional gastrointestinal disorder characterized by symptoms such as postprandial fullness, early satiety, upper abdominal pain, and heartburn (Ford et al., 2020). Its diagnosis is based on the Rome IV diagnostic criteria, which were introduced in Alonso-Bermejo et al. (2022). The pathogenesis of FD is multifaceted, involving abnormalities in gastrointestinal motility, visceral hypersensitivity, dysregulation of the brain-gut axis, and psychological factors (Labanski et al., 2020; Sayuk and Gyawali, 2020). In fact, so far, only acid suppression treatment with proton pump inhibitors has been proven to be beneficial for FD (Wauters et al., 2021a). The presence of anxiety, a common comorbidity in FD patients, further complicates the therapeutic process and increases the difficulty of achieving a cure (Esterita et al., 2021). Recent advancements in the understanding of the brain-gut axis have revealed a close association between gut microbiota dysbiosis and symptoms of FD with anxiety (Rupp and Stengel, 2022). This has led us to hypothesize that probiotics, which can modulate the gut microbiota, may be beneficial in treating FD with anxiety, potentially through improvements in intestinal mucosal permeability.

In recent years, the role of the brain-gut axis in the pathogenesis of FD has garnered increasing attention. The brain-gut axis is a complex bidirectional regulatory system that involves intricate connections between the central nervous system and the gut (Mayer et al., 2022). This bidirectional communication not only affects gastrointestinal function but is also closely related to emotions and psychological states. Studies have shown that FD patients often suffer from psychological disorders such as anxiety, which may exacerbate gastrointestinal symptoms through the brain-gut axis (Mikocka-Walus et al., 2023). Additionally, gut microbiota dysbiosis and increased intestinal mucosal permeability may impact the central nervous system via the brain-gut axis, further worsening the symptoms of FD patients (Tait and Sayuk, 2021).

Intestinal mucosal permeability refers to the degree to which the intestinal mucosa allows various antigens and toxins to pass through. Under normal conditions, the intestinal mucosal barrier effectively prevents harmful substances from entering the bloodstream. However, when intestinal mucosal permeability is increased, bacterial endotoxins (Lipopolysaccharide (LPS)), inflammatory factors (such as Interleukin-1 (IL-1), Interleukin-6 (IL-6), Tumor Necrosis Factor-*α* (TNF-α)), and intestinal mucosal permeability markers [such as Zonulin, Human Diamine Oxidase (DAO), Human Intestinal Fatty Acid Binding Protein (I-FABP)] (Seethaler et al., 2021; Fasano, 2020) can enter the bloodstream, triggering systemic inflammatory responses that subsequently affect gastrointestinal function. Zonulin is an endogenous regulatory protein that primarily acts on the tight junctions between intestinal epithelial cells. An increase in Zonulin release is typically associated with intestinal barrier dysfunction, and elevated Zonulin levels are linked to increased intestinal permeability. When the intestinal mucosal barrier is compromised, DAO rapidly translocates from the intestinal lumen across the mucosa into the peripheral blood. A decrease in DAO activity is commonly associated with increased intestinal permeability, In healthy individuals, serum DAO levels are relatively stable, but they significantly rise when the intestinal mucosal barrier is damaged (Huang et al., 2021). When the small intestinal mucosal tissue is injured, I-FABP is quickly released into the circulation, and an increase in its plasma concentration can serve as an early marker of small intestinal damage (Funaoka et al., 2010).

In fact, the pathophysiological link between FD and anxiety is not only related to gut microbiota dysbiosis and the release of intestinal inflammatory factors, but also to the role of neurotransmitters (Bosman et al., 2023; Hassamal, 2023). Cortisol, a stress hormone, significantly increases in levels during states of anxiety (Patel et al., 2024). Cortisol not only affects emotional regulation but may also exacerbate the symptoms of FD by influencing the autonomic nervous function of the gastrointestinal tract and the composition of the gut microbiota (Ravenda et al., 2025). Elevated cortisol levels can lead to enhanced sympathetic nervous activity in the gastrointestinal tract, inhibiting gastrointestinal motility and increasing visceral hypersensitivity. GABA (*γ*-Aminobutyric Acid): GABA is an important inhibitory neurotransmitter that plays a key role in regulating anxiety and gastrointestinal function (Chen et al., 2024). A decrease in GABA levels is associated with worsening anxiety symptoms and may also affect gastrointestinal motility and sensory function (Qin et al., 2022).

Anxiety is a common comorbid psychological disorder in FD patients and plays a significant role in the development of FD. A large-scale survey study from Guangzhou Medical University in 2015 showed that the proportion of refractory FD patients with comorbid anxiety was 61.5% (Jiang et al., 2015). Anxiety not only exacerbates gastrointestinal symptoms but may also affect treatment outcomes and quality of life. Studies have shown that patients with anxiety may have more pronounced increases in intestinal mucosal permeability, which may be one of the potential mechanisms underlying the exacerbation of FD symptoms by anxiety (Stevens et al., 2018). A decade-long survey based on the Swedish population revealed that the likelihood of having FD with anxiety was eight times higher than in those without anxiety (Aro et al., 2015). Therefore, exploring the differences between FD patients with and without anxiety, as well as the role of intestinal mucosal permeability, is of significant importance for understanding the pathogenesis of FD and optimizing treatment strategies.

Probiotics, as a means of regulating gut microbiota and improving intestinal mucosal permeability, have been widely used in the treatment of FD patients (Ritchie and Romanuk, 2012). Probiotics can alleviate the symptoms of FD patients through mechanisms such as regulating gut microbiota balance, enhancing intestinal mucosal barrier function, and inhibiting inflammatory responses (Tziatzios et al., 2023). However, the therapeutic effect of probiotics on FD patients with anxiety and its relationship with intestinal mucosal permeability remain unclear. This study aims to compare the baseline characteristics and intestinal mucosal permeability indicators of FD patients with and without anxiety, explore the impact of probiotic intervention on symptoms and anxiety scores in both groups, and investigate the relationship between changes in intestinal mucosal permeability indicators and symptom relief and anxiety improvement.

We hypothesize that probiotic intervention will significantly improve symptoms and anxiety scores in FD patients with anxiety, and that this improvement will be closely related to changes in intestinal mucosal permeability indicators. We hope that this study will further clarify whether probiotics are beneficial for the improvement of symptoms in FD patients with anxiety and whether this benefit is related to changes in intestinal mucosal permeability. This will help to further reveal the potential role of intestinal mucosal permeability and enhance our understanding of the role of the brain-gut axis.

## Methods

### Study design

This study is a randomized controlled trial, included a total of 282 patients diagnosed with FD from July 2023 to February 2025 at the Affiliated Sanming First Hospital, Fujian Medical University (China), with patients divided into two groups: those with FD accompanied by anxiety and those with FD without anxiety. Within each group, participants were randomly assigned to either the intervention group or the control group using computer-generated random numbers. The intervention group received conventional treatment plus oral probiotics, while the control group received conventional treatment alone. Conventional treatment refers to acid suppression, gastric mucosal protection, and prokinetic therapy for FD. The intervention period lasted one and a half months. The study was approved by the Ethics Committee of Sanming First Hospital (Ming Yi Lun 2023 No. 73) and supported by the Natural Science Foundation of Fujian Province (Grant No. 2024J011500). It was registered (ChiCTR2300077847) before inclusion of the first participant and its protocol has been publicly available. All patients underwent gastroscopy and abdominal CT scans. All participants provided informed consent.

### Participants

**Figure 1:**
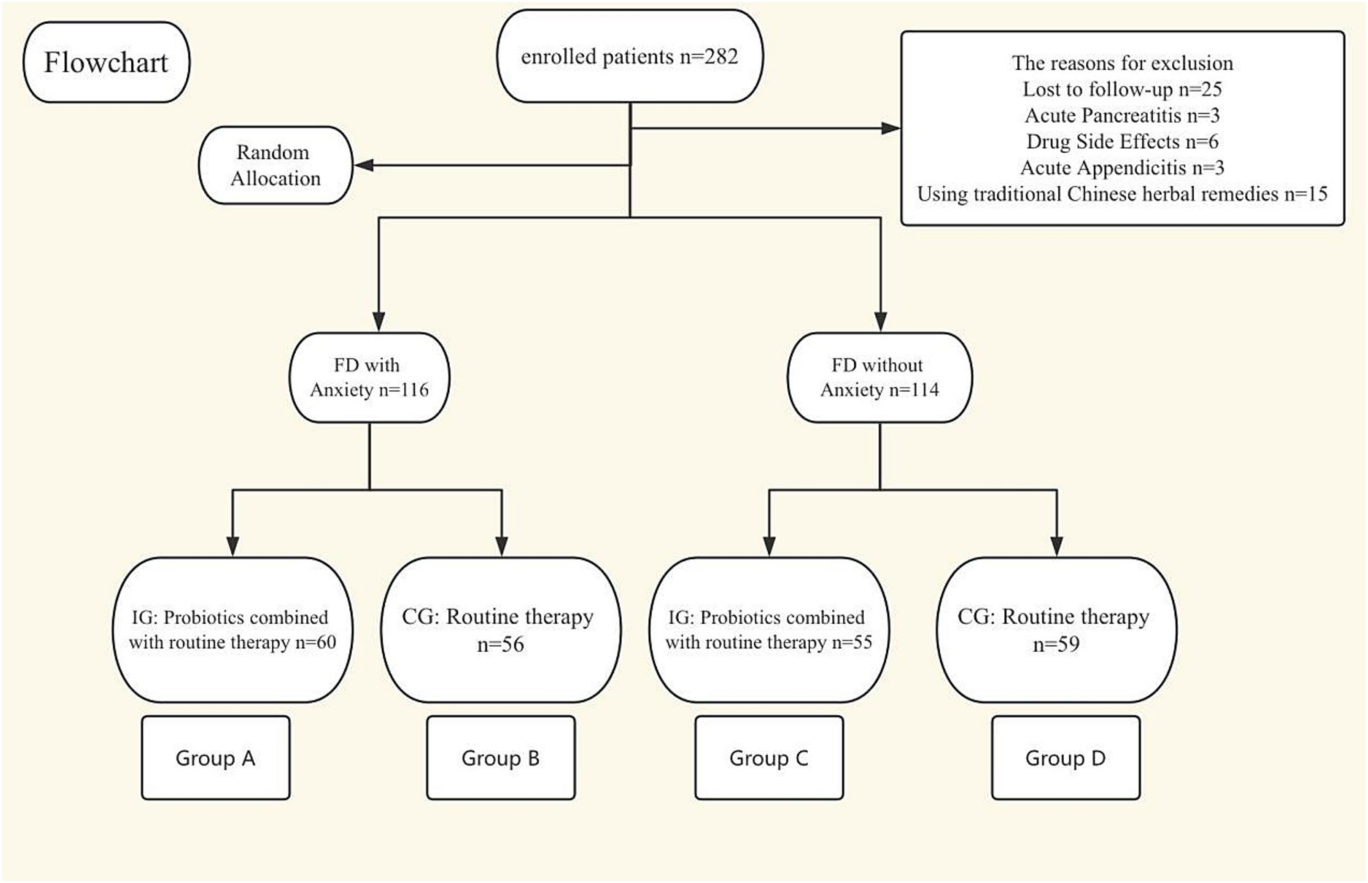
Flow diagram.

Inclusion criteria for this study were as follows: (1) Age between 18 and 80 years old; (2) Patients diagnosed with FD; (3) The diagnostic criteria for FD were based on the Rome IV criteria: Presence of one or more of the following symptoms: Bothersome postprandial fullness; Bothersome epigastric pain; Bothersome epigastric burning. The criteria must have been met for the past 3 months, with symptoms occurring for at least 6 months prior to diagnosis. Additionally, the patient must meet the criteria for Postprandial Distress Syndrome (PDS) and/or Epigastric Pain Syndrome (EPS). Exclusion criteria: (1) Have taken relevant medications in the last 1 month (antibiotics, probiotics, herbs, anti-anxiety medications); (2) Detection of organic diseases by gastroscopy (such as peptic ulcer, gastric cancer, etc.); (3) Presence of primary diseases or space-occupying lesions in the liver, gallbladder, pancreas, spleen, etc., or other intestinal and abdominal manifestations of diseases such as biliary ascariasis and typhoid fever; (4) Acute onset, such as acute appendicitis, gastrointestinal perforation, intussusception, intestinal obstruction, etc.; (5) Presence of connective tissue diseases and their complications or severe organ diseases; (6) Hamilton Anxiety Scale score exceeding 31 points (or if we believe that intervention by a psychiatrist is necessary); (7) Allergy to the study medication or being pregnant or breastfeeding; 8. Loss to follow-up (Figure 1).

### Scale rating measurement

The assessment of all scales was conducted by specially trained personnel, with each scale’s rating completed within 10 min.

#### The Hamilton Anxiety Scale (HAMA)

HAMA (Thompson, 2015) is used to assess the severity of anxiety symptoms in individuals. It consists of 14 items, with each item scored on a scale from 0 to 4 based on the severity of the symptom: 0: The symptom is not present. (1) The symptom is mild and has minimal impact on life and activities. (2) The symptom is present and noticeable but does not affect life and activities. (3) The symptom is severe, requires additional intervention, and may have already affected life and activities. (4) The symptom is very severe and has a significant impact on life and activities. The total score ranges from 0 to 56, with higher scores indicating more severe anxiety. 0–7: No anxiety; 8–17: Mild anxiety; 18–25: Moderate anxiety; 25–30: Severe anxiety; Above 31: Very severe anxiety.

#### Patient Assessment of Gastrointestinal Disorders-Symptom Severity Index (PAGI-SYM)

The PAGI-SYM (Rentz et al., 2004) is a self-assessment scale used to evaluate the severity of symptoms in patients with upper gastrointestinal disorders. It consists of 20 items divided into six subscales, which assess the following symptom clusters: Heartburn/regurgitation; Fullness/early satiety; Nausea/vomiting; Bloating; Upper abdominal pain; Lower abdominal painEach item is scored on a scale from 0 (no symptoms) to 5 (very severe). The total score is 100, with higher scores indicating more severe symptoms.

### Detection of serum inflammatory factors and intestinal mucosal permeability markers

Blood samples were collected from patients in the morning after an 8-h fast and centrifuged within 1 h of collection using a centrifuge (Anhui USTC Zonkia Science Instruments Co., China, Model LC-4016, Instrument No. 0311000107) at 3,000 rpm for 10 min. The supernatant serum was then divided into 200-μL aliquots. The serum samples were stored in a freezer at −80°C. For testing, the samples were equilibrated at room temperature for 20 min, followed by enzyme-linked immunosorbent assays (ELISA). All reagents were thoroughly mixed before testing. The ELISA kits used were as follows: IL-1 Kit (Item No. ZK-H233); IL-6 Kit (Item No. ZK-H238, Lot NO.202503); TNF-*α*, (Lot NO.202503) Kit (Item No. ZK-H064, Lot NO.202503); I-FABP Kit (Item No. ZK-H051, Lot NO.202503); DAO Kit (Item No. ZK-H384, Lot NO.202503); Human Zonulin Kit (Item No. ZK-H4119, Lot NO.202503); LPS Kit (Item No. ZK-H1337, Lot NO.202503). All kits and Enzyme Labeler (Model mb-580) were purchased from Ziker Biological Technology Co., Ltd., Shenzhen, China. Levels of inflammatory mediators and markers of intestinal mucosal permeability in the serum were measured before and after treatment.

### Probiotics and compliance

The intervention group received 0.5 g of *Bacillus licheniformis* capsules three times daily and 0.42 g of *Bifidobacterium* triple viable capsules twice daily, in addition to conventional acid suppression (Proton Pump Inhibitors (PPIs): Rabeprazole 20 mg, taken orally once daily), mucosal protection (Almagate susp 15 mL, taken orally three times daily), and prokinetic therapy (Itopride hydrochloride 50 mg, taken orally three times daily). The doses of the probiotics used in this study were based on previous clinical trials and recommendations from the manufacturers (Wallace et al., 2023; Zhang et al., 2020; Haghighat et al., 2021). The control group received the same doses of placebo capsules along with the conventional acid suppression, mucosal protection, and prokinetic therapy. The *Bacillus licheniformis* spore capsules (each capsule contains 250 million live bacteria) are produced by Zhejiang Jingsheng Pharmaceutical Co., Ltd. The *Bifidobacterium* triple viable bacteria preparation is a compound preparation (each gram contains at least 1.0 × 10^6^ CFU of *Bifidobacterium longum*, at least 1.0 × 10^6^ CFU of *Lactobacillus acidophilus*, and at least 1.0 × 10^6^ CFU of *Enterococcus faecalis*), produced by Jincheng Haisi Pharmaceutical Co., Ltd. The placebo has the same appearance but does not contain live bacteria. The contents of both the probiotics and the placebo are packaged in bags with similar appearance, and it is difficult to distinguish them in terms of color, taste, and smell. The bagged products should be stored in a dry place below 25°C and away from direct sunlight. Study participants were also instructed to do so. Adverse drug reactions were recorded during the study period. We accept a compliance rate of 80 to 100% through regular follow-ups conducted via telephone.

### Statistical analysis

The Kolmogorov–Smirnov test was used to analyze whether the measurement data conformed to a normal distribution (*p* > 0.05). Quantitative measurement data are expressed as the mean and standard deviation (x ± S) if they conformed to a normal distribution. In this case, *t* tests were used to compare the two groups, and differences between the two groups before and after treatment were compared using paired t tests. Measurement data are presented as the median and interquartile range [M (P25, P75)] if they did not conform to a normal distribution. In this case, the MannWhitney U test (rank and inspection) was used to compare the two groups, and differences between the two groups before and after treatment were compared using the Wilcoxon signed-rank sum test. Qualitative data are presented as the frequency and rate. Differences in categorical variables were assessed by the *χ*^2^ test or Fisher’s exact test (when the expected count was <5) and Pearson’s *χ*^2^ test. Differences between multiple groups were compared using the Kruskal-Wallis test. A two-tailed *p* value of <0.05 was considered statistically significant. The factors influencing the improvement of anxiety scores before and after treatment were analyzed using linear regression analysis. SPSS (version 26.0) was used for statistical analysis.

## Result

### Baseline information of the patients

A total of 282 patients were enrolled in this study, of whom 230 completed the study. Twenty-five patients were lost to follow-up and excluded during the study period. Three patients were excluded due to acute pancreatitis. Six patients were excluded due to adverse drug reactions. Three patients were excluded due to acute appendicitis. Fifteen patients were excluded for using traditional Chinese medicine during the study (see flow diagram).

Baseline data of patients with FD with and without anxiety were analyzed. The results showed that there was a statistically significant difference in the gender composition between the two groups, with a higher proportion of females in the FD with anxiety group compared to the FD without anxiety group (*χ*^2^ = 14.62, *p* < 0.01). There was no statistically significant difference in age (*Z* = −0.72, *p* = 0.469). There were statistically significant differences in the blood test indicators IL-1, IL-6, TNF-*α*, LPS, Zonulin, DAO, and I-FABP between the two groups (*p* < 0.01). Additionally, there were statistically significant differences in the scale scores of PAGI-SYM and HAMA between the two groups (*p* < 0.01) (Table 1).

**Table 1:** Baseline data of patients with FD accompanied by anxiety and those with FD without anxiety.

Characteristic	FD with anxiety	FD without anxiety	*χ*^2^/*Z*	*p*-value
Sex, *n*	Male 40人	Male 68人	*χ*^2^ = 14.62	< 0.001
	Female 76人	Female 46人		
Age, years	54.5 (49.0, 60.8)	56 (50, 61)	*Z* = −0.72	0.469
IL-1	153.8 (146.0, 160.8)	80.1 (61.2, 97.3)	*Z* = 13.11	< 0.001
IL-6	105.2 (86.4, 124.9)	95.2 (80.6, 114.0)	*Z* = 2.42	0.015
TNF-a	87.4 (66.7, 95.8)	75.9 (65.3, 88.5)	*Z* = 3.05	0.002
LPS	1299.6 (1135.9, 1432.8)	725.4 (587.4, 880.6)	*Z* = 12.73	< 0.001
Zuonlin	204.3 (177.0, 218.2)	98.4 (75.7, 125.3)	*Z* = 12.43	< 0.001
DAO	26.9 (22.0, 29.4)	15.9 (13.5, 18.5)	*Z* = 10.05	< 0.001
I-FABP	1436.3 (1183.1, 1622.6)	816.1 (669.2, 1096.4)	*Z* = 13.15	< 0.001
HAMA	20 (17, 23)	3 (2,4)	*Z* = 13.14	< 0.001
PAGI-SYM	46 (40, 55)	19.5 (13,23)	*Z* = 13.08	< 0.001

HAMA, The Hamilton Anxiety Scale; PAGI-SYM, Patient Assessment of Gastrointestinal Disorders-Symptom Severity Index.

FD with anxiety were divided into an intervention group and a control group. We compared the blood test indicators before and after the intervention for each subgroup. For data that did not meet the normal distribution, we used the non-parametric *U* test. The results showed that in the intervention group, there were statistically significant differences in IL-1 (*Z* = 9.51, *p* < 0.001), IL-6 (*Z* = 6.38, *p* < 0.001), TNF-*α* (*Z* = 6.28, *p* < 0.001), LPS (*Z* = 6.30, *p* < 0.001), Zuonlin (*Z* = 6.42, *p* < 0.001) and DAO (*Z* = 6.73, *p* < 0.001) before and after treatment. There were no statistically significant differences in I-FABP (*Z* = −0.74, *p* = 0.46) before and after treatment. In terms of scale scores, there were statistically significant difference in PAGI-SYM (*Z* = 6.740, *p* < 0.001), HAMA (*Z* = 6.671, *p* < 0.001) before and after treatment. In the control group, there were statistically significant difference in IL-6 (*Z* = 5.17, *p* < 0.001) before and after treatment. There were no statistically significant differences in IL-1 (*Z* = 0.73, *p* = 0.47), TNF-*α* (*Z* = −0.74, *p* = 0.46), LPS (*Z* = 0.81, *p* = 0.42), Zonulin (*Z* = 0.10, *p* = 0.92), DAO (*Z* = 0.86, *p* = 0.40) and I-FABP (*Z* = 1.27, *p* = 0.205) before and after treatment. In terms of scale scores, there was a statistically significant difference in PAGI-SYM (*Z* = 5.61, *p* < 0.001) before and after treatment. There was no statistically significant difference in HAMA (*Z* = 1.89, *p* = 0.06) before and after treatment. We conducted a visual analysis of the blood test indicators (Table 2).

**Table 2:** Changes in indicators before and after treatment in the FD with anxiety group.

Characteristic	FD with anxiety *n* = 60 (IG)		z	*p*	FD with anxiety *n* = 56(CG)		z	*p*
	Before treatment	after treatment			Before treatment	after treatment		
IL-1	150.8 (143.0, 158.0)	119.7 (102.4, 135.9)	9.51	**	156.8 (149.0, 163.7)	154.6 (138.2, 171.4)	0.73	0.47
IL-6	109.3 (94.9, 130.6)	75.9 (60.1, 95.1)	6.38	**	100.6 (78.0, 121.1)	69.5 (57.0, 88.3)	5.17	**
TNF-a	94.3 (90.2, 101.3)	71.1 (61.3, 87.7)	6.28	**	67.3 (48.3, 85.2)	68.1 (49.9, 84.3)	−0.74	0.46
LPS	1298.6 (1204.0, 1382.4)	1024.4 (907.8, 1164.6)	6.30	**	1299.6 (1063.3, 1474.4)	1250.7 (996.4, 1427.0)	0.81	0.42
Zuonlin	208.9 (197.8, 217.7)	161.3 (135.6, 184.9)	6.42	**	191.9 (143.6, 221.0)	182.8 (151.2, 227.8)	0.10	0.92
DAO	29.3 (27.5, 30.4)	21.9 (18.4, 24.8)	6.73	**	22.7 (16.0, 25.3)	19.4 (13.1, 26.0)	0.86	0.40
I-FABP	1413.2 (1159.1, 1626.0)	1416.3 (1209.0, 1637.8)	−0.74	0.46	1462.5 (1252.5, 1614.5)	1353.9 (1126.9, 1596.4)	1.29	0.20
PAGI-SYM	50 (41.0, 55.8)	34.0 (26.3, 41.0)	6.740	**	46.5 (42.0, 56.0)	43.0 (36.3, 52.5)	5.61	**
HAMA	21.0 (17.3, 25.0)	15.5 (13.3, 20.0)	6.671	**	20.0 (16.3, 23.0)	20.0 (16.3, 22.0)	1.89	0.06

HAMA, The Hamilton Anxiety Scale; PAGI-SYM, Patient Assessment of Gastrointestinal Disorders-Symptom Severity Index; IG, Intervention Group; CG, Control Group **: *p*<0.001.

FD without anxiety were divided into an intervention group and a control group. We compared the blood test indicators before and after the intervention for each subgroup. For data that did not meet the normal distribution, we used the non-parametric *U* test. The results showed that in the intervention group, there were statistically significant differences in IL-6 (*Z* = 2.09, *p* = 0.04) and TNF-*α* (*Z* = 4.15, *p* = 0.000) before and after treatment. There were no statistically significant differences in IL-1 (*Z* = −1.59, *p* = 0.11), LPS (*Z* = 0.85, *p* = 0.40), Zonulin (*Z* = −1.19, *p* = 0.23), DAO (*Z* = 0.31, *p* = 0.76), and I-FABP (*Z* = −1.75, *p* = 0.08) before and after treatment. In terms of scale scores, there was a statistically significant difference in PAGI-SYM before and after treatment (*Z* = 6.72, *p* < 0.001). In the control group, there was a statistically significant difference in LPS (*Z* = −2.66, *p* = 0.008) before and after treatment. There were no statistically significant differences in IL-1 (*Z* = −1.36, *p* = 0.17), IL-6 (*Z* = −1.69, *p* = 0.09), TNF-α (*Z* = −1.01, *p* = 0.31), Zonulin (*Z* = −0.58, *p* = 0.56), DAO (*Z* = 0.17, *p* = 0.86), and I-FABP (*Z* = −0.46, *p* = 0.65) before and after treatment. In terms of scale scores, there was a statistically significant difference in PAGI-SYM before and after treatment (*Z* = 6.681, *p* < 0.001) (Table 3).

**Table 3:** Changes in indicators before and after treatment in the FD without anxiety group.

Characteristic	FD without anxiety *n* = 55 (IG)		*z*	*p*	FD without anxiety *n* = 59(CG)		*z*	*p*
	Before treatment	after treatment			Before treatment	after treatment		
IL-1	82.5 (65.9, 100.7)	87.4 (71.0, 109.8)	−1.59	0.11	80.1 (58.6, 96.2)	81.6 (70.8, 103.3)	−1.36	0.17
IL-6	108.6 (90.2, 127.5)	101.6 (75.1, 124.7)	2.09	0.04	93.9 (81.3, 114.6)	103.1 (85.1, 126.1)	−1.69	0.09
TNF-a	78.1 (69.9, 88.6)	63.1 (44.5, 84.5)	4.15	0.00	70.3 (62.4, 88.3)	71.0 (63.6, 79.0)	−1.01	0.31
LPS	748.3 (530.1, 910.8)	655.7 (536.3, 828.8)	0.85	0.40	719.2 (591.5, 814.6)	780.6 (652.7, 940.5)	−2.66	0.008
Zuonlin	105.6 (79.2, 126.0)	110.1 (90.8, 126.0)	−1.19	0.23	94.4 (72.4, 125.9)	104.5 (77.2, 120.8)	−0.58	0.56
DAO	15.3 (13.3, 19.1)	15.3 (11.8, 19.0)	0.31	0.76	16.2 (13.6, 17.7)	15.3 (11.5, 19.3)	0.17	0.86
I-FABP	871.5 (663.6, 1097.2)	965.9 (754.6, 1212.0)	−1.75	0.08	816.6 (678. 2,1128.7)	834.4 (644.0, 1061.0)	−0.46	0.65
PAGI-SYM	19.5 (13.0, 24.8)	11.5 (8.0, 16.0)	6.72	< 0.001	20.0 (13.3, 23.0)	13.0 (11.0, 17.0)	6.681	< 0.001

PAGI-SYM, Patient Assessment of Gastrointestinal Disorders-Symptom Severity Index; IG, Intervention Group; CG, Control Group.

We divided patients with FD with and without anxiety into two subgroups each. The intervention group of FD with anxiety was labeled as Group A, and the control group of FD with anxiety was labeled as Group B. The intervention group of FD without anxiety was labeled as Group C, and the control group of FD without anxiety was labeled as Group D. We conducted chi-square tests to analyze the differences between these subgroups. We defined a reduction of more than 20% in the PAGI-SYM symptom scale score as an improvement in symptoms. The results showed that there were statistically significant differences in PAGI-SYM symptom relief between Group A and Group B (*χ*^2^ = 10.20, *p* = 0.001); between Group A and Group C (*χ*^2^ = 23.30, *p* < 0.001); between Group A and Group D (*χ*^2^ = 10.30, *p* = 0.001); between Group B and Group C (*χ*^2^ = 54.22, *p* < 0.001); and between Group B and Group D (*χ*^2^ = 35.85, *p* < 0.001). However, there was no statistically significant difference in PAGI-SYM symptom relief between Group C and Group D (χ^2^ = 3.26, *p* = 0.071) (Table 4).

**Table 4:** Analysis of differences in PAGI-SYM symptom relief among the subgroups.

Characteristic	Effective	Ineffective	Total	*χ* ^2^	*p*-value
A	20	40	60	10.20	0.001
B	5	51	56		
Total	25	91	116		
A	20	40	60	23.30	< 0.001
C	43	12	55		
Total	63	52	115		
A	20	40	60	10.30	0.001
D	37	22	59		
Total	57	62	119		
B	5	51	56	54.22	< 0.001
C	43	12	55		
Total	48	63	111		
B	5	51	56	35.85	< 0.001
D	37	22	59		
Total	42	73	115		
C	43	12	55	3.26	0.071
D	37	22	59		
Total	80	34	114		

A, FD with Anxiety (IG); B, FD with Anxiety (CG); C, FD without Anxiety (IG); D, FD without Anxiety (CG).

In this study, patients whose PAGI-SYM scores decreased by more than 20% after treatment compared to before treatment were defined as having symptom relief. We established a binary logistic regression analysis model, with symptom relief defined as 1 and no relief defined as 0. The independent variables included intestinal mucosal permeability markers, gender, age, and whether there was probiotic intervention. The results showed that a smaller I-FABP (OR = 0.999, *p* = 0.036), LPS (OR = 0.998, *p* = 0.013) and probiotic intervention (OR = 3.206, *p* = 0.002) were statistically significant for symptom relief. The other indicators showed no statistical difference in terms of symptom relief (Table 5).

**Table 5:** Analysis of the impact of baseline intestinal mucosal permeability on the improvement of upper gastrointestinal symptoms.

Characteristic	B	SE	Wald	*p*-value	OR	LCL (95% CI)	UCL (95% CI)
LPS	−0.002	0.001	6.109	0.013	0.998	0.996	1.000
Zuonlin	−0.007	0.005	2.198	0.853	0.138	0.984	1.002
DAO	0.093	0.040	5.291	0.021	1.097	1.014	1.188
I-FABP	−0.001	0.001	4.377	0.036	0.999	0.998	1.000
Sex	0.084	0.325	0.067	0.796	0.919	0.486	1.739
Age	0.011	0.023	0.214	0.644	1.011	0.966	1.058
Probiotic	1.165	0.369	9.993	0.002	3.206	1.557	6.602

LCL, Lower Confidence Limit; UCL, Upper Confidence Limit.

We conducted a linear regression analysis on the improvement of anxiety scores in patients with FD and anxiety, incorporating changes in intestinal mucosal permeability markers as independent variables. The results showed that greater changes in LPS (*t* = 2.014, *p* = 0.046), Zonulin (*t* = 2.463, *p* = 0.015), and DAO (*t* = 3.054, *p* = 0.003) were associated with more significant improvements in anxiety scores. In contrast, changes in I-FABP (*t* = −0.412, *p* = 0.681) were not statistically significant in relation to the improvement of anxiety scores (Table 6).

**Table 6:** The impact of intestinal permeability levels on anxiety relief.

Characteristic	B	SE	*t*	*p*-value	LCL (95% CI)	UCL (95% CI)
LPS	0.002	0.001	2.014	0.046	0.000	0.004
Zuonlin	0.011	0.005	2.463	0.015	0.002	0.021
DAO	0.114	0.037	3.054	0.003	0.040	0.188
I-FABP	0.000	0.001	−0.412	0.681	−0.002	0.001

LCL, Lower Confidence Limit; UCL, Upper Confidence Limit.

## Discussion

The main findings of this study are as follows: 1. Patients with FD and anxiety have higher levels of intestinal permeability markers (LPS, Zonulin, DAO, and I-FABP) compared to those with FD without anxiety (*p* < 0.01); 2. Probiotics are effective in improving HAMA scores in patients with FD and anxiety (*p* < 0.001); 3. After probiotic intervention, the symptom relief rate of PAGI-SYM in patients with FD and anxiety is lower than that in patients with FD without anxiety; 4. In the group of patients with FD and anxiety, the improvement of anxiety is correlated with changes in LPS, Zonulin, and DAO.

### Clinical significance of intestinal mucosal permeability in FD with anxiety

Previous studies have already confirmed that patients with FD have increased intestinal mucosal permeability (Wu et al., 2021; Wauters et al., 2020). The increase in intestinal mucosal permeability leads to the release of more inflammatory factors, which can exacerbate the symptoms of FD (Hari et al., 2022). The observed significant differences in various markers of intestinal mucosal permeability (LPS, Zonulin, DAO, and I-FABP) between FD patients with and without anxiety suggest that increased intestinal mucosal permeability may play a pivotal role in the exacerbation of symptoms in FD patients with anxiety. The results of this study indicate that the levels of intestinal mucosal permeability may be even higher in patients with FD when anxiety is comorbid. In our study, we found that without probiotic intervention, the decrease in PAGI-SYM scores was significantly lower in the FD with anxiety group compared to the FD without anxiety group. This indicates that anxiety plays a certain interfering role in the alleviation of FD symptoms. However, despite both groups receiving probiotic intervention, the relief of FD symptoms in the FD with anxiety group was almost the same as that in the FD without anxiety group.

Neilan et al. (2014) have used the lactulose/mannitol (L/M) ratio, which reflects small intestinal permeability, to compare differences between patients with FD and control groups. The results indicated that there were no statistically significant differences between the two groups. The study suggested that this might be because the inflammation in FD could also be limited to a sufficiently short segment of the upper gastrointestinal tract, such that the L/M ratio, which is affected by the permeability of the entire small intestine, may not be a sensitive enough indicator. This seems inconsistent with our study, in our analysis of factors affecting the relief of FD symptoms, we found that lower levels of I-FABP, an indicator of small intestinal permeability, were associated with easier relief of FD symptoms, suggesting that the relief of FD symptoms may be related to the permeability of the small intestinal mucosa.

This aligns with the broader understanding that the brain-gut axis, a bidirectional communication system between the central nervous system and the gastrointestinal tract, is significantly disrupted in these patients (Labanski et al., 2020). The increased permeability allows bacterial endotoxins and inflammatory mediators to enter the systemic circulation, triggering a cascade of inflammatory responses that can further impair gastrointestinal function and contribute to the persistence of symptoms. In fact, the results of this study also show that in patients with FD and anxiety, the improvement of anxiety is related to the improvement of intestinal mucosal permeability (LPS, Zonulin, DAO), while changes in I-FABP do not play a role in the improvement of anxiety. We speculate that this may be because I-FABP mainly reflects the permeability of the small intestine, and the intestinal permeability or the low-grade chronic inflammation in patients with anxiety is mainly not manifested in the small intestine. This also supports the role of the gut-brain axis in the relationship between intestinal mucosal permeability and anxiety.

### Clinical efficacy of probiotics in functional dyspepsia patients with anxiety

Increasing evidence suggests that the use of probiotics for the treatment of FD is a highly promising approach (Ford et al., 2014). The mechanisms of action of probiotics are likely to be multifactorial (Hosseini et al., 2012), they can restore microbial symbiosis by eliminating pathogenic bacteria, mediate epithelial barrier permeability, alter visceral hypersensitivity, exert local and systemic anti-inflammatory effects, and modulate intestinal motility, thereby influencing the severity of FD symptoms (Wauters et al., 2020; Ford et al., 2014). The results of this study indicate that probiotics are highly beneficial for the relief of upper gastrointestinal symptoms and the improvement of anxiety in patients with FD and anxiety.

Moreover, the improvement in HAMA scores suggests that this effect is associated with changes in intestinal mucosal permeability, as evidenced by the significant correlation between improvements in anxiety levels and changes in LPS (*t* = 2.014, *p* = 0.046), Zonulin (*t* = 2.463, *p* = 0.015), and DAO (*t* = 3.054, *p* = 0.003) levels. In a 2017 study, Igarashi et al. (2017) not only documented changes in the microbial profiles of gastric fluid in patients with FD, but also observed a beneficial shift in the microbial composition of gastric fluid following probiotic treatment, aligning more closely with that of healthy control volunteers. The efficacy of probiotic treatment for FD has also been documented by Drago et al. (2021) who reported a significant decrease in symptom prevalence among patients with PDS treated solely with probiotics. Wauters et al. (2021b) conducted a randomized controlled study involving 68 patients, where the experimental group was given probiotic capsules twice daily, and the placebo group was administered capsules containing maltodextrin (which contains no symbiotic bacteria). The results indicated that the clinical symptom scores of the experimental group with FD significantly decreased (*p* < 0.05), whereas no significant changes were observed in the placebo group. Rahmani et al. (2020) conducted a 4-week randomized, double-blind, placebo-controlled clinical trial involving 125 patients with FD. In this study, 65 participants received probiotics (*Lactobacillus reuteri*), while the remaining participants received a placebo. The results showed that, compared to baseline, all FD-related variables, such as the frequency, severity, and duration of pain, significantly decreased by the end of week 4. It can be seen that some studies have focused on the improvement of FD symptoms by probiotics, but very few studies have been conducted on the improvement of anxiety in FD by probiotics. The results of this study suggest that probiotics are not only effective in improving FD symptoms, but also in alleviating anxiety, which may be related to the improvement of intestinal mucosal permeability.

## Limitations and future directions

The results of this study support the efficacy of probiotic treatment for FD with anxiety, but there are still limitations in the study. First, this study did not analyze the strains of probiotics. In the future, treatment should be individualized based on different bacterial communities and populations to make the treatment more targeted. Second, this study was a single-center study. In the future, research should be conducted in more centers to make the study results more reliable. Third, there was a lack of analysis of the various components of the gut microbiota. For example, there was no comprehensive analysis of the gut microbiota of patients before and after treatment. Future studies could use genome sequencing to fully understand the changes in the gut microbiota and their correlation with intestinal mucosal permeability markers, upper gastrointestinal symptoms, and anxiety. In the future, personalized probiotic treatment plans could also be developed based on patients’ baseline characteristics (such as gut microbiota composition, inflammation levels, and degree of anxiety). Personalized treatment is expected to improve treatment outcomes and reduce unnecessary side effects.

## Conclusion

In conclusion, this study provides compelling evidence that probiotics can significantly improve symptoms and anxiety scores in FD patients with anxiety, with the therapeutic effects being closely related to changes in intestinal mucosal permeability. The findings highlight the potential of probiotics as an effective therapeutic strategy for managing FD with anxiety and underscore the importance of targeting intestinal mucosal permeability in the treatment of these patients. Future research should focus on elucidating the precise mechanisms through which probiotics modulate intestinal mucosal permeability and exploring the long-term clinical outcomes of probiotic interventions in this patient population.

## Data Availability

The raw data supporting the conclusions of this article will be made available by the authors, without undue reservation.
